# New Roads Open Up for Implementing Immunotherapy in Mesothelioma

**DOI:** 10.1155/2012/927240

**Published:** 2012-06-24

**Authors:** R. Cornelissen, M. E. Heuvers, A. P. Maat, R. W. Hendriks, H. C. Hoogsteden, J. G. J. V. Aerts, J. P. J. J. Hegmans

**Affiliations:** ^1^Department of Pulmonary Medicine, Erasmus Medical Center-Daniel den Hoed Cancer Center, P.O. Box 2040, 3000 CA Rotterdam, The Netherlands; ^2^Department of Thoracic Surgery, Erasmus Medical Center-Daniel den Hoed Cancer Center, P.O. Box 2040, 3000 CA Rotterdam, The Netherlands

## Abstract

Treatment options for malignant mesothelioma are limited, and the results with conventional therapies have been rather disappointing to this date. Chemotherapy is the only evidence-based treatment for mesothelioma patients in good clinical condition, with an increase in median survival of only 2 months. Therefore, there is urgent need for a different approach to battle this malignancy. As chronic inflammation precedes mesothelioma, the immune system plays a key role in the initiation of this type of tumour. Also, many immunological cell types can be found within the tumour at different stages of the disease. However, mesothelioma cells can evade the surveillance capacity of the immune system. They build a protective tumour microenvironment to harness themselves against the immune system's attacks, in which they even abuse immune cells to act against the antitumour immune response. In our opinion, modulating the immune system simultaneously with the targeting of mesothelioma tumour cells might prove to be a superior treatment. However, this strategy is challenging since the tumour microenvironment possesses numerous forms of defence strategies. In this paper, we will discuss the interplay between immunological cells that can either inhibit or stimulate tumour growth and the challenges associated with immunotherapy. We will provide possible strategies and discuss opportunities to overcome these problems.

## 1. Introduction

Links between cancer and inflammation were first noted by Rudolf Virchow in 1863, on observations that tumours often arose at sites of chronic inflammation and that inflammatory cells were present in biopsy samples from tumours [1]. In a severe combined immunodeficiency (SCID) mouse xenograft model, it has recently been shown that inflammation precedes the development of human malignant mesotheliomas [2]. Also, epidemiological studies have revealed that chronic inflammation caused by chemical and physical agents, autoimmune and by inflammatory reactions of uncertain aetiology, predisposes for certain forms of cancer [3, 4]. Recently our group demonstrated a significantly shorter survival in patients with lung cancer in subjects with a history of pulmonary tuberculosis than patients without tuberculosis [5], revealing even a more complex interplay between inflammation and cancer. Increasing evidence indicates that the inflammation-cancer connection is not only restricted to the initiation of the cancer process, since all types of clinically manifested cancers appear to have an active inflammatory component in their microenvironment. These experimental findings and clinical observations have led to cancer-related inflammation being acknowledged as an important hallmark of cancer [6].

## 2. Immunooncology

### 2.1. Tumour-Immune Surveillance

Old, Klein, and others investigated murine tumour transplantation models and showed that the immune system of healthy recipient mice was able to distinguish transformed malignant cells from normal cells [7, 8]. Even preceding these publications, Frank MacFarlane Burnet and Lewis Thomas formulated their cancer immunosurveillance hypothesis: “It is by no means inconceivable that small accumulations of tumour cells may develop and because of their possession of new antigenic potentialities provoke an effective immunological reaction with regression of the tumour and no clinical hint of its existence" [9]. At that time this hypothesis was controversial; however, with the current knowledge and ongoing research, it is apparent their premise seems to be correct because there is strong evidence from animal studies that cells of the immune system carry out surveillance and can eliminate nascent tumours [10].

Tumour-associated antigens (TAAs) are antigens acquired by tumour cells in the process of neoplastic transformation that can elicit a specific immune response by the host. It is known that several immunological cell types are involved in the recognition and destruction of tumours during early stages of development. These include cells and factors of the innate immune system, including macrophages, neutrophils, complement components, *γδ* T cells, natural killer (NK) cells, NKT cells and certain cytokines (IL-12, IFN-*γ*) and cells of the adaptive immune system, including B lymphocytes, helper T cells (Th cells), and cytotoxic T lymphocytes (CTLs).

TAAs need to be presented to the cells of the adaptive immune system. Dendritic cells (DCs) are widely acknowledged for their potent antigen presenting capacity and play a key role in the initiation of this adaptive immune response by activation and modulation of lymphocyte subsets [11]. DCs originate from bone marrow precursor cells and are found at low frequencies in peripheral tissues where they maintain an immature phenotype and search their surroundings for foreign substances. Immunogenic TAAs are secreted or shed by tumour cells or released when tumour cells die and can be taken up by DCs or other antigen presenting cells (APCs). Upon encountering an antigen, DCs mature and migrate to regional draining lymphoid organs. The captured antigen is processed and presented by major histocompatibility complex (MHC) class I and class II molecules on their cell membrane leading to the activation of antigen-specific lymphocytes. This results in antibody production by B lymphocytes and tumour-specific CTLs to assist the innate immune responses in the killing of tumour cells.

### 2.2. Tumour-Immune Escape

Increasing evidence reveals that when tumours progress in time, tumour cells undergo changes to escape immune surveillance. This process encompasses three phases: elimination, equilibrium, and escape. During the first phase, tumour cells have to escape the immune surveillance to survive. Then these surviving tumour cells can enter the equilibrium state, in which there is equilibrium between tumour growth and tumour killing by cells of the immune system. In this stage, tumours can persist for years without progressing to more severe tumour stages. However, during this period, tumour cells may undergo mutations caused by their genetic instability, potentially generating variants that can escape the immune system, by either evading the induction of an immune response or by inhibiting antitumour responses via a variety of immune suppressive mechanisms.

### 2.3. Immune Suppressive Mechanisms

The tumour immune escape mechanism can be greatly enhanced by the induction of an immune suppressive tumour microenvironment. In this microenvironment, inflammatory cells and molecules have a major influence on cancer progress. Effective adaptive immune responses are suppressed through the activation of several pathways. For example, the differentiation and activation of dendritic cells, which are the key initiators of adaptive immune responses, are inhibited by signals (such as IL-10 and VEGF) present in the tumour microenvironment. In addition, tumours, peripheral blood, and lymph nodes contain increased amounts of regulatory T cells (Tregs), which suppress both the adaptive and innate immune responses [12]. Also, a heterogeneous population of myeloid-derived suppressor cells (MDSCs) are induced in tumour-bearing hosts; these cells, as well as tumour-associated macrophages (TAMs or M2 macrophages), are potent suppressors of antitumour immunity. Not only do MDSCs and TAMs suppress the antitumour response, but they also assist the malignant behaviour of tumour cells by secreting cytokines, growth factors, matrix-degrading enzymes, and proteases, which promote tumour progression or enhance metastasis.

**Figure 1:**
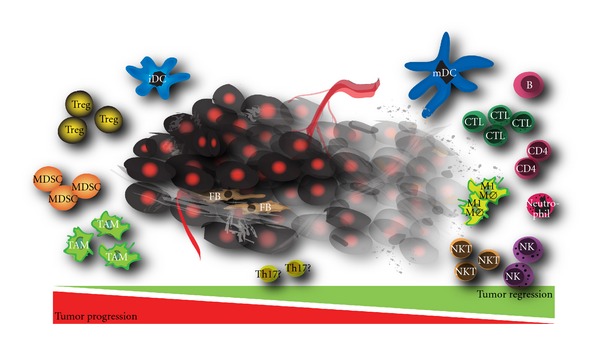
Interplay between immunological cells that inhibit tumour growth on the right of the tumour and cells that aid in tumour progression on the left. (Tumour is depicted as black cells with a red nucleus in the middle.) iDC: immature dendritic cell, Treg: regulatory T cell, MDSC: myeloid-derived suppressor cell, TAM: tumour-associated macrophage, mDC: mature dendritic cell, B: B cell lymphocyte, CTL: cytotoxic T lymphocyte, M1 MØ: M1 macrophage, NK(T): natural killer (T) cell, Th17: helper T lymphocyte 17, FB: fibroblast.

In conclusion, immune cells can either protect the host against cancer development or promote the emergence of tumours with reduced immunogenicity leading to a complex interplay of tumour growth and tumour regression mechanisms (Figure 1) [13].

## 3. Immunotherapy

Cancer immunotherapy attempts to activate or enhance the antitumour effects of the immune system of the patient, or it may assist in the capabilities of the immune system to fight cancer. Multiple approaches for immunotherapy have been developed over the years, and many are in various stages of (pre-)clinical research. Immunotherapy can be divided into two main categories: passive and active immunotherapy [14].

### 3.1. Passive Immunotherapy

Passive immunotherapy makes use of *in vitro* produced immunologic effectors that are capable of influencing tumour cell growth. The most common form of passive immunotherapy is called monoclonal antibody therapy. It consists of humanized monoclonal antibodies that are investigated in several human malignancies. Monoclonal antibodies can target cells directly [15] or indirectly. Monoclonal antibodies are also used as immune modulators to inhibit immune suppressive molecules/cells or activate immune stimulatory molecules. Efficacy of this approach can sometimes be enhanced by linking a toxin to these antibodies (e.g., radionucleotides or anticancer drugs).

In mesothelioma, preclinical studies targeting mesothelin with immunotoxins CAT-5001 (formerly SS1P) and amatuximab (previously known as MORab-009) were promising [16–18] and therefore progressed to clinical trials. CAT-5001, administered to mesothelioma patients, among other cancer types, showed only modest clinical responses [17, 18]. Amatuximab failed to demonstrate any radiological responses in a phase I trial in mesothelioma and other cancer types [19]; however preclinical studies demonstrated significant antitumour efficacy using combination of amatuximab and chemotherapy treatment [20] justifying a multicenter phase II clinical trial utilizing cisplatin/pemetrexed with amatuximab in mesothelioma patients. This trial has been completed and results are expected soon. More recently a phase I study of SS1(dsFv)PE38, a recombinant antimesothelin immunotoxin, was commenced which is ongoing at this moment (ClinicalTrials.gov Identifier: NCT00575770). 

Another method of passive immunotherapy uses adaptive transfer of (autologous or allogeneic) antigen-specific effector cells (like T cells and NK cells) that can be expanded and/or activated *ex vivo* and subsequently administered to the patient to attack the tumour [21]. This approach showed the potential to reconstitute host immunity against pathogens, like Epstein-Barr virus (EBV) in immune suppressed patients, but more importantly also provides evidence that adaptive T cell transfers can prevent the induction of EBV-associated lymphomas [22]. This led to the concept that antigen-specific T cell transfer can be used as an antitumour therapy to eradicate established tumours. The approach of adaptive T cell transfer to eradicate malignancies is challenging [23].

### 3.2. Active Immunotherapy

Active immunotherapeutic approaches aim at inducing or boosting immune effector cells *in vivo *against tumour cells, through the administration of immune mediators capable of activating the immune system.

Several cytokines are capable of activating and recruiting specific immune cells that can enhance antitumour immunity (e.g., IL-2, IL-12, IL-15, TNF-*α*, GM-CSF). These cytokines can be used as single agent or in combination with other immunotherapeutic strategies.

Defined TAA epitopes have been used to vaccinate cancer patients [24]; however this approach is limited by the relatively low number of identified specific peptides and by the requirement of MHC typing. By using the whole TAA protein for immunization, the need of peptide identification can be circumvented. These proteins can be taken up by APCs and endogenously processed into epitopes for presentation to T cells. Adjuvants need to be added to induce APCs activation and avoid tolerance induction [25].

DNA sequences coding for specific TAAs can be directly injected into the skin. DNA then needs to be taken up, transcribed into mRNA, translated into a protein, and processed into peptides by APCs.

In mesothelioma, the TAA's mesothelin and Wilms tumour-1 (WT-1) are highly expressed and thought to be physiologically relevant to this tumour type [26]. In the Memorial Sloan-Kettering Cancer Center a phase I peptide vaccination clinical trial in mesothelioma patients is ongoing (ClinicalTrials.gov Identifier: NCT01265433). In these patients, inoculation with WT-1 peptide elicited WT-1-specific CD4 and CD8 T-cell responses, with minimal toxicity [26]. TroVax has been shown to stimulate an immune response to a particular protein widely found on mesothelioma cells called 5T4; a clinical trial testing the effectiveness of TroVax is currently active in the Wales Cancer Trials Unit (ClinicalTrials.gov Identifier: NCT01569919).

An important restriction of this method is the relatively inefficient delivery into APCs. Viruses engineered to express TAAs can be injected directly into the patient. The virus then infects the host cell, leading to cell death and presentation of antigenic epitopes to the immune system. A wide variety of viral vectors are available. Currently, a trial using intrapleural administration of a vaccine with a measles virus strain is performed at the Mayo Clinic (ClinicalTrials.gov Identifier: NCT01503177). However there are concerns regarding the immune dominance of viral antigens over TAAs, resulting in a strong anti-virus response leading to virus eradication and attenuation of the antitumour immune response [27].

DCs have emerged as the most powerful initiators of immune responses. In the natural activation of the adaptive immune system against tumour cells, DCs play a crucial role since they are capable to engulf tumour antigens and activate lymphocytes in an antigen-specific manner. Therefore, the application of DCs to therapeutic cancer vaccines has been prompted [28].

The research group of Dr. Robinson published a very interesting trial, in which they used an autologous tumour lysate vaccine that was manufactured from surgically resected mesothelioma material and administered subcutaneously together with granulocyte-macrophage colony stimulating factor (GM-CSF). GM-CSF facilitates APCs recruitment and survival *in vivo* which in turn may generate tumour-specific immunity after uptake of the TAA from the lysate. Twenty-two patients were enrolled onto this trial. Of these, five developed positive delayed type hypersensitivity skin tests, and five showed evidence of altered antibody specificities by western blotting, proving that GM-CSF could induce tumour-specific immunity, both cellular and humoral responses. 32% of the patients developed at least one type of anti-MM immune response. Furthermore, the therapy was safe and was associated with stable disease; however no major tumour regressions were observed [29].

While this study showed potential for GM-CSF as immunotherapeutical approach, *in vivo* stimulation of APCs is also a very attractive method. Sipuleucel-T is an active cellular immunotherapy consisting of autologous peripheral-blood mononuclear cells (PBMCs), including APCs. Recently, Kantoff et al. published a phase III trail where they used *ex vivo* activated Sipuleucel-T with a recombinant fusion protein (PA2024). PA2024 consists of a prostate antigen, prostatic acid phosphatase that is fused to GM-CSF, an immune-cell activator. Sipuleucel-T prolonged survival among men with asymptomatic or minimally symptomatic metastatic castration-resistant prostate cancer [30], providing evidence for cell-based immunotherapeutic agents in solid tumours.

In mesothelioma, the source of the TAA for DC loading remains a critical issue that will determine the efficacy of the DC-based vaccination. A careful identification and characterization of antigenic epitopes is needed when peptides want to be used. However, the ideal source of TAAs may be the tumour itself, since it expresses all the TAAs that can be targeted.

Incubating DCs with dead tumour cells (necrotic or apoptotic cells), as was shown in a pioneering article by the research group of Dr. Gregoire, DCs were exposed to a full array of antigenic peptides that rapidly gain access to both MHC Class I (cross-presentation) and MHC Class II pathways, therefore leading to a diversified immune response involving cytotoxic T lymphocytes (CTLs) as well as CD4+ T cells. In their paper they successfully demonstrated *in vitro* culture and antigen loading in a human mesothelioma model, resulting in a specific CTL response [31].

One of the advantages of an *ex vivo* culture model is that DCs can be generated in large amounts and pulsed with tumour antigens under optimal conditions. In mesothelioma, we previously investigated the effect of DC-based immunotherapy on the outgrowth of mesothelioma in a murine model [32]. We established that DC-based immunotherapy induced strong tumour-specific CTLs responses leading to prolonged survival in mice. The efficacy of immunotherapy was dependent on the tumour load; most beneficial effects were established at early stages of tumour development.

**Figure 2:**
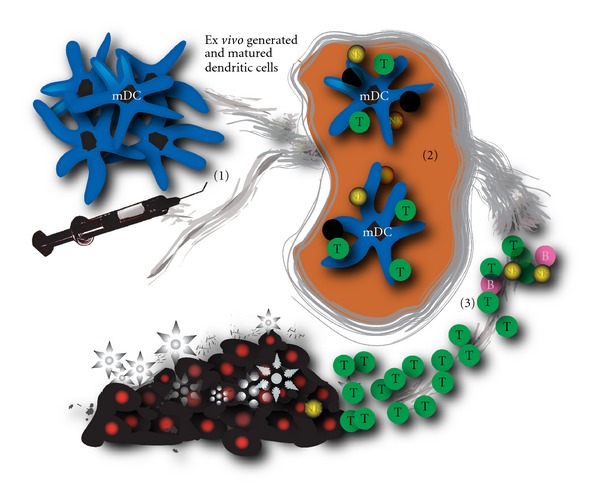
Schematic drawing showing the administration of *ex vivo* cultured mature dendritic cells into a patient (1), resulting in antigen presentation in the lymph node (2) and a specific antitumour cytotoxic antitumour response (3). Tumour cells are depicted as dark cells.

On the basis of these preclinical animal studies, we have performed the first clinical trial in which autologous tumour lysate-pulsed DCs were administrated in mesothelioma patients [33]. Patients were eligible for the study when sufficient tumour cells could be obtained from pleural effusion or tumour biopsy material at the time of diagnosis. DC-immunotherapy was planned after completion of the cytoreductive therapy provided that during chemotherapy no major side effects occurred and there was no progressive disease. Patients received three immunizations with mature DCs, loaded with autologous tumour lysate. Each immunization, consisting of 50 × 10*e*6 cells, was administered intradermally and intravenously (Figure 2). Overall, the vaccination regimen with loaded DCs was well tolerated, and a successful immune reaction was induced by the DC vaccinations.

The University Hospital of Antwerp has started a similar protocol in mesothelioma and several other solid tumours but is using WT-1 as antigen loading for the DCs (ClinicalTrials.gov Identifier: NCT01291420), circumventing the need for patient's tumour material.

Another method to load DCs is to make use of measles-virus-infected mesothelioma cells. It was shown that this method induced spontaneous DC maturation and that priming of autologous T cells by DCs loaded with measles-virus-infected mesothelioma cells led to a significant proliferation of tumor-specific CD8 T cells [34].

## 4. Improving Immunotherapy

While immunotherapy was proven safe and feasible, it has not established its place yet in mesothelioma treatment. Partly, this is due to the presence of immunosuppressive cells in peripheral blood, lymphoid organs, and within the tumour environment that hamper immunotherapeutic treatments. Several strategies have been performed or are currently tested that target the immunosuppressive cells aiming to improve the efficacy of immunotherapy. In the following sections, we will focus on three populations of suppressive cells, the MDSCs, Tregs, and TAMs, that are increased in most cancer patients. It is becoming increasingly clear that these populations contribute to the impaired antitumour responses frequently observed in cancer patients. Therefore, combating immunosuppression through modulation of these cell types will be an important key to increase the efficacy of immunotherapy and should lead to better prognosis for cancer patients.

### 4.1. Myeloid-Derived Suppressor Cells

MDSCs are a heterogeneous population of bone-marrow-derived myeloid cells, comprising of immature monocytes/macrophages, granulocytes, and DCs at different stages of differentiation [35]. A subset of MDSCs, mononuclear MDSCs (MO-MDSCs), is mainly found at the tumour site while polymorph nuclear MDSCs (PMN-MDSCs) subset is found in blood, lymphoid organs, and at the tumour site. They express a number of surface markers, that are on themselves not unique but in combination can define MDSCs. MDSCs are increased in cancer patients, and it is anticipated that they play a suppressive role during the innate and adaptive immune responses to cancer but have also been described in the course of other pathologic processes such as thermal injury, various infectious diseases, sepsis, trauma, after bone marrow transplantation, and in some autoimmune disorders.

Activation of MDSCs not only requires tumour-derived factors (e.g., tumour-derived prostaglandin E2 (PGE2)) but also IFN-*γ* produced by T cells and factors secreted by tumour stromal cells (like IL-1*β*, IL-4, IL-6, IL-10, IL-13). Activation of cytokine receptors on MDSCs leads to activation of STAT-signalling pathways, resulting in the production of immune suppressive substances (like TGF-*β*, reactive oxygen species (ROS), and nitric oxide synthetase (NOS)).

MDSCs can inhibit the antitumour immune response in several ways.

- MDSCs are capable of producing reactive oxygen species (ROS) and peroxynitrite, which is responsible for most of the adverse effects on T cells, linked to ROS. Changes caused by nitration of the T cell receptor make T cells incapable of interacting with the MHC complex on APCs, which is necessary to obtain T cell-specific stimulation [36, 37].
- MDSCs can inhibit the antitumour response in an antigen nonspecific manner by the high expression of the enzyme inducible nitric oxide synthetase (iNOS), leading to the generation of NO. NO can suppress T cell function though various mechanisms including the inhibition of the cell signalling pathways and inducing DNA damage to T cells.
- Arginase-I activity by MDSCs depletes L-arginine from the environment, contributing to the induction of T cell tolerance by downregulating the CD3*ζ*-chain expression of the T cell receptor [38, 39].
- MDSCs block T-cell activation by sequestering cysteine and thus limiting the availability of the essential amino acid cysteine [40].
- MDSCs can inhibit T cell proliferation by producing IL-10 and TGF-*β* [41].
- Antitumour cells, like NK- and NKT-cells, can be inhibited by MDSCs via TGF-*β*1-dependent mechanisms. MDSC can bind to the TGF-*β* receptor on target cells via membrane-bound TGF-*β*, leading to activation of intracellular pathways resulting in downregulation of NK-specific receptors [41].
- The plasma membrane expression of enzyme ADAM17 on MDSCs cleaves L-selectin on naïve T cells, decreasing their ability to home to sites where they could be activated [42].
- MDSCs can indirectly enhance immune suppression via the induction of Tregs [43–45].
- MDSCs differentiate under certain biological conditions into mature functionally competent macrophages or to DCs influencing tumoural responses [46].

### 4.2. Targeting MDSCs

Both gemcitabine and 5-fluorouracil (5FU) have shown to be selectively cytotoxic on MDSC in murine tumour models [47]. The treatment of tumour-bearing mice with 5FU led to a decrease in the number of MDSC in the spleens and tumour beds of animals whereas no significant effect on T cells, NK cells, DCs, or B cells was noted. 5FU showed a superior efficacy over gemcitabine to deplete MDSC and selectively induced MDSC apoptotic cell death [47].

Gene expression profile analysis of multiple tumour types identified SCF (c-kit ligand) as a candidate tumour factor involved in MDSC accumulation. Inhibiting c-kit using the tyrosine kinase inhibitor sunitinib resulted in a decrease of the number of MDSC and Treg in advanced tumour-bearing animals [49].

The production of ROS by MDSCs, which is responsible for most of the adverse effects on T cells, is highly depending upon cyclooxygenase-2 (COX-2) enzyme activity [50]. The inducible COX-2 enzyme is essential in the biosynthesis of prostaglandins. Celecoxib is a selective COX-2 inhibitor. Therefore, we investigated the effect of celecoxib treatment on the four MDSC subsets that were identified in the spleen of tumour-bearing mice [51]. When combining DC-based immunotherapy and celecoxib treatment, a significant improvement of the immunotherapy was seen in comparison to no or single modality treatment. Treatment of tumour-bearing mice with dietary celecoxib prevented the local and systemic expansion of all MDSC subtypes, and also their suppressive function was impaired. At the National Cancer Institute, allogeneic tumour cell vaccine is combined with celecoxib and metronomic oral cyclophosphamide as adjuvants in thoracic malignancies (ClinicalTrials.gov Identifier: NCT01143545); the rationale for using cyclophosphamide is discussed further in this paper.

### 4.3. Tumour-Associated Macrophages

Macrophages are a major component of the leukocyte infiltrate in the tumour microenvironment [52] and have even been described as key orchestrators of cancer-related inflammation [53]. Classically activated (M1) macrophages, following exposure to IFN-*γ*, have antitumour activity and tissue-destructive activity. In response to IL-4 or IL-13, macrophages undergo alternative (M2) activation. M2 macrophages are oriented to tissue repair, tissue remodelling, and immune regulation. TAMs generally have the phenotype and functions similar to M2 macrophages and display a defective NF-*κ*B activation in response to different proinflammatory signals [54].

TAM recruitment in tumours is mediated by several cytokines, of which CCL2 seems to be the main player; other chemokines involved in monocyte recruitment are CCL5, CCL7, CXCL8, and CXCL12, as well as cytokines such as VEGF, PDGF, and the growth factor M-CSF [53]. It has been shown that MO-MDSCs are capable of differentiating towards TAMs. Therefore, similar recruitment factors are described that contribute to the infiltration of TAMs and MDSCs into tumour tissue [55]. In addition, dynamic changes of the tumour microenvironment occur during the transition from early neoplastic events toward advanced tumour stages resulting in local hypoxia, low glucose level, and low pH. These events in the tumour microphysiology drive the switch from a M1 macrophage toward the M2 type.

TAMs are able to suppress the adoptive immune response through various mechanisms and contribute to angiogenesis and tumour invasiveness.

- TAMs are able to produce immune suppressive cytokines, like CCL17, CCL18, CCL22, IL-1*β*, IL-6, IL-10, and TGF-*β*. IL-10 in combination with IL-6 can lead to upregulation of molecules in TAMs, which are implicated in suppression of tumour-specific T cell immunity [56].
- TAMs express the enzyme indoleamine 2,3-dioxygenase (IDO), a well-known suppressor of T cell activation. IDO catalyzes the catabolism of tryptophan, an essential amino acid acquired for T cell activation [57].
- TAMs contribute to immune suppression via indirect ways. Secretion of CCL18 leads to recruitment of native T cells. Attraction of naive T cells into the tumour microenvironment is likely to induce T cell anergy [58]. Besides CCL18, CCL17 and CCL22 are abundantly expressed. These cytokines interact with CCR4 receptor expressed by Tregs and induce T-helper 2 polarization [59]. Via expression of VEGF, TAMs can block antigen uptake by APCs and attract MDSCs, which can function as TAM precursors but are also actively suppressing T cell function. MDSCs are depending on prostaglandin E2 (PGE2) for their function. PGE2 is secreted by many types of cancer; however, TAMs are also capable of producing PGE2 and therefore assist MDSC function [60].
- In tumour stroma, TAMs produce matrix metalloproteases (MMP) and other proteases, leading to degradation of the extracellular matrix. During this process, several cytokines, chemokines, and growth factors are released from the matrix that promotes and facilitates endothelial cell survival and migration and thereby enhances angiogenesis [61].
- Besides indirect mechanisms, angiogenesis is also directly stimulated by TAMs. TAMs can produce proangiogenic factors like vascular endothelial growth factor (VEGF), transforming growth factor (TGF)-*β*, and platelet-derived growth factors (PDGFs). The release of these factors leads to the neovascularisation, especially in hypoxic regions within the tumour [53, 62].
- In addition to angiogenesis, TAMs are also strongly involved in lymphangiogenesis, a process mediated by a number of factors including VEGF-C and VEGF-D via VEGFR3 [53].
- Outside the scope of the tumour microenvironment, but a pivotal step in general tumour biology, TAMs cooperate on tumour dissemination by promoting invasion characteristics. One of the main factors involved significantly is TNF-*β*: coculture of neoplastic cells with macrophages enhances invasiveness of malignant cells through TNF-dependent MMP induction by macrophages [53].

### 4.4. Targeting TAMs

There is accumulating evidence supporting the hypothesis that effects on TAMs may contribute to the antitumour effect of bisphosphonates [63]. We investigated the effect of zoledronic acid (ZA) in mesothelioma-inoculated mice. Our data showed that the addition of ZA to macrophage-inducing culture conditions significantly inhibits the upregulation of F4/80, MHCII, and CD11c. In addition, these data reveal that adding tumour supernatant leads to polarization of the macrophage phenotype towards M2 subtype and that ZA can prevent this polarization *in vitro*, leading to a significant reduction in the CD206 expression on macrophages cultured in the presence of ZA. *In vivo*, however, no significant differences on tumour progression and survival could be observed between untreated mice and mice treated with ZA, because the reduction in TAMs was associated with an increase in MDSC [64].

IL-6 stimulates tumour macrophage infiltration in ovarian cancer, and recently it has been shown that this action can be inhibited by the neutralizing anti-IL-6 antibody siltuximab in preclinical and clinical studies [65].

A recent study revealed that activation of macrophages by the infusion of antibodies against CD40 may induce macrophage-mediated tumour regression in 30% of cases in both a mouse model for pancreatic cancer and in patients with pancreatic cancer [66, 67].

Since TGF-*β* is responsible for skin tumour infiltration by macrophages enabling the tumours to escape immune destruction [68], TGF-*β* seems to be a major player in the formation of the suppressive tumour microenvironment. Blockade of TGF-*β* has been shown to enhance tumour vaccine efficacy, but at this moment the exact mechanism has not been unravelled yet [69]. Since CCL2 plays a major role in the recruitment of TAMs, anti-CCL2 would be a logical step in preventing this recruitment. However, it seems that anti-CCL2 does not prevent the influx of TAMs [70]; this could be due to the inability to reach an adequate dosage of anti-CCL2 in the tumour microenvironment to counteract the influx of TAMs.

### 4.5. Regulatory T Cells

Tregs are a population of CD4+ T cells with a central role in the prevention of autoimmunity and the promotion of tolerance via their suppressive function on a broad repertoire of cellular targets [71]. Characteristic of human Tregs is the expression of CD25 (IL-2 receptor-*α* chain), forkhead box P3 (Foxp3) transcription factor, glucocorticoid-induced TNF-receptor-related-protein (GITR), lymphocyte activation gene-3 (LAG-3), cytotoxic T-lymphocyte-associated antigen 4 (CTLA4), and a downregulation of CD127 (IL-7R); however, all these markers are not truly Treg specific [72]. Tregs can be divided into natural Tregs and adaptive Tregs. Natural Tregs are important in the suppression of autoreactive T cells that slip through the selection processes, and therefore natural Tregs maintain peripheral tolerance against self-antigens preventing autoimmunity. In humans, these cells represent 2–5% of total circulating CD4+ T cells in peripheral blood [73]. Adaptive Tregs arise from naive T cells and are triggered by suboptimal antigen stimulation and stimulation with TGF-*β*. Adaptive Tregs can be subdivided into IL-10 secreting Tregs type I (Tr1 cells), TGF-*β* producing Tregs (Th3 cells), or IL-35 secreting Tregs (iTr35 cells). These cells are characterized by the secretion of immune suppressive cytokines directly inhibiting T cells and converting DCs into suppressive APCs [74]. This contagious spread of suppressive capacity, mainly mediated by IL-35 from Tregs, to other T cells is called infectious tolerance [75].

Tregs infiltrate human cancers, and their prevalence in tumour-infiltrating lymphocytes is much higher than their proportion in peripheral blood, constituting 20% or more of tumour-infiltrating CD4+ lymphocytes [76]. Elevated levels of Tregs have been identified in blood of cancer patients, compared with normal individuals, and their presence predicts for poor survival [77]. In mesothelioma patients, elevated levels of Tregs have also been identified in pleural fluid, with a clear patient-to-patient variability [78].

Natural Tregs are derived in the thymus and migrate into the periphery. It has been proposed that Tregs need to be activated and/or expanded from periphery and bone marrow if needed. Since 25% of CD4+ T cells in the bone marrow function as Tregs, it has been suggested that the bone marrow plays an active role in humoral and cellular immune regulation.

TAA-specific Tregs accumulate in the peripheral lymphoid organs and at the tumour side. However TAA-specific Tregs are also found in the bone marrow, suggesting that after activation Tregs can migrate back to the bone marrow and induce T cell tolerance before these cells enter the circulation [79]. Although exact mechanisms are not fully explored, it has been shown that CCR4+ (receptor for CCL22) Tregs migrate toward tumour microenvironments expressing CCL22 [12]. Also CD62L and CCR7 have been described as important homing markers on Tregs [80]. CD62L is critical for the migration of Tregs to draining lymph nodes. CCR7 is expressed by a majority of Tregs and is essential in homing to lymphoid organs and microenvironments expressing CCL19 (the ligand for CCR7) [81].

As MDCSs and TAMs, Tregs have several pathways that limit antitumour responses.

- Direct cell-cell interaction between Tregs and target cells is important for tolerance induction by Tregs [82]. These target cells include CD4+ and CD8+ effector cells, B cells, NK, T cells, DCs, and monocytes/macrophages. The cell-cell binding leads to apoptosis by activation of programmed cell death ligands (PDLs), the release of perforin [83] and granzyme-A or B [36] and by reducing the proliferation through upregulation of intracellular cyclic AMP [84, 85].
- Tregs produce themselves or induce other cells to secrete immunosuppressive cytokines such as IL-10, IL-35, and TGF-*β* to blunt immune responses [86], but also other molecules produced by Tregs like carbon monoxide [87] and galectins [88] are reported to play roles in suppression. However, the relative importance of the individual inhibitory factors is dependent on the target disease and experimental model.
- Tregs can inhibit antitumour effector NK and NK T cells via membrane-bound TGF-*β* [89]. The binding of membrane-bound TGF-*β* on Tregs to TGF-*β*-receptor on target cells leads to the activation of intracellular pathways, which eventually leads to the downregulation of the NKG2D-receptor on NK and NKT cells.
- CTLA4+ Tregs induce the expression of indoleamine 2,3-dioxygenase (IDO) in APCs, a potent regulatory molecule mediating the catabolism of the essential amino acid tryptophan into the proapoptotic kynurenine, which is toxic to neighbouring T cells [90].
- Tregs are forming aggregates around DCs to prevent contact between DCs and T cells and in this way disturb the induction of the adaptive immune response by preventing proper antigen presentation [91, 92].
- Treg aggregation leads to decreased upregulation of CD80 and CD86 on immature DCs and downregulation of these molecules on mature DCs [93]. These phenomena are antigen specific and dependent on lymphocyte function-associated antigen 1 (LFA-1) and CTL-associated protein 4 (CTLA-4) [22].
- Tregs induce B7-H4 expression by APCs, a member of the B7 family that negatively regulates T-cell responses [94].
- Expression of both ectoenzymes CD39 and CD173 on Tregs can hydrolyse pericellular ATP/AMP into the cAMP or the immunosuppressive nucleoside adenosine [95].
- Binding of lymphocyte activation gene 3 (LAG3) on Tregs to the MHC class II molecules expressed on immature DC suppresses DC maturation [96].
- Activated Tregs, which express more high-affinity IL-2R than conventional T cells, may absorb IL-2 from the microenvironment and therefore starve effector T cells that need IL-2 to survive [97].

However, none of these mechanisms can explain all aspects of suppression. It is probable that various combinations of several mechanisms are operating, depending on the milieu and the type of immune responses.

### 4.6. Targeting Tregs

Owing to the significant role of Tregs in the failure of immune surveillance and immunotherapy, many attempts to deplete Tregs or inhibit their function in cancer patients have been studied. Many of the strategies to reduce Tregs target CD25, which makes up the alpha subunit of the IL-2R, that is present on the surface of Tregs and activated cells. An engineered recombinant fusion protein of IL-2 and diphtheria toxin (denileukin diftitox [Ontak]) and other CD25-directed immunotoxins (daclizumab, LMB-2, RFT5-SMPT-dgA) have been investigated for Treg depletion, which seems to kill selectively lymphocytes expressing the IL-2 receptor. However, early human trials have not proven that this approach results in tumour regression and have shown that these strategies may not adequately deplete Foxp3+ Tregs and may also deplete antitumour effector cells [98–101]. Other possible approaches to reduce immunosuppression of Tregs are via CTLA-4 blockade (e.g., ipilimumab) [102, 103], anti-GITR agonism [104], and vaccination against Foxp3 [105], and some other suggested approaches, such as the inhibition of IDO, TGF-*β*, ectonucleotidase (expressed by Tregs and generates immunosuppressive adenosine), or the activation of other agents such as OX40 or Toll-like receptor 8 have not yet proven to be beneficial. IL-7 administration was shown to increase T cell numbers and decrease the Treg fraction in humans [106]; on the contrary, other reports have shown that IL-7 leads to the development of Tregs [107, 108]. In conclusion, there are many conflicting results in abrogating the action of Tregs, and thus it is unclear which approach holds promise for cancer treatment.

Low-dose cyclophosphamide (CTX) prevents the development and functionality of the Tregs [109]; the mechanism behind this effect, however, is not completely understood. We investigated the effect of CTX on immune suppression, and the combination of CTX and DC-based immunotherapy was studied in a murine MM model [110]. Our data showed that metronomic administration of low-dose CTX has a strong immune-modulating effect *in vivo*. This is currently tested in a clinical trial in mesothelioma patients (ClinicalTrials.gov Identifier: NCT01241682). Tregs can be significantly reduced in mice with antimurine CCL2/CCL12 monoclonal antibodies, resulting in significant reductions in Treg cells in the spleens and tumours. Using these antibodies, the tumour microenvironment was also drastically altered. This resulted in a significant improvement of immunotherapy [69]. Sorafenib has been proven cytotoxic for Tregs, although the pathway is not fully understood. Sorafenib treatment is associated with a decrease in frequency of Treg cells without influencing the function of peripheral immune effector cells [111]. Recently, p300 was found to be an important target for modulation of host Foxp3+ Treg functions, and an inhibition of p300 using a small molecule inhibitor, C646 (p300i), impaired Foxp3 acetylation and inhibited Treg function [112].

### 4.7. Immune-Adjuvant Therapies

An alternative approach to immunotherapy is to enhance the intrinsic activity of the immune system. In this field, ipilimumab was proven to be active in metastatic melanoma [113]. Ipilimumab is a monoclonal antibody against cytotoxic T-lymphocyte antigen (CTLA)-4. It is normally expressed at low levels on the surface of naïve effector T cells but is upregulated on the cell surface when there is a long-lasting and strong stimulus via the T cell receptor (TCR). CTLA-4 then competes with CD28 for CD80/CD86 on APCs, effectively shutting off TCR signalling, and thereby serves as a physiologic “brake” on the activated immune system [114]. Ipilimumab prevents this feedback inhibition, resulting in an unabated immune response against the tumour. The side effects of this therapy, however, can be significant due to the downregulation of tolerance to patient's own normal tissue, and colitis is often seen in patients [115]. In mesothelioma, preclinical models have been well described, and a phase II trial is currently ongoing in Italy [26].

Other preclinical approaches are the Toll-like receptor (TLR) ligands to activate DCs [116] or TLR7 agonist to induce systemic, CD8+ T-cell, and type-I IFN antitumour responses [117].

## 5. Need for Revising Response Evaluation in Immunotherapy

Immunotherapy represents a new class of agents in the treatment of mesothelioma. As seen for Sipuleucel-T in prostate cancer and ipilimumab in melanoma, improvement in the overall survival of patients was seen; however, the agents did not change initial disease progression. Even a transient worsening of disease manifested either by progression of known lesions or the appearance of new lesions can be seen, before disease stabilizes or tumour regresses.

**Figure 3:**
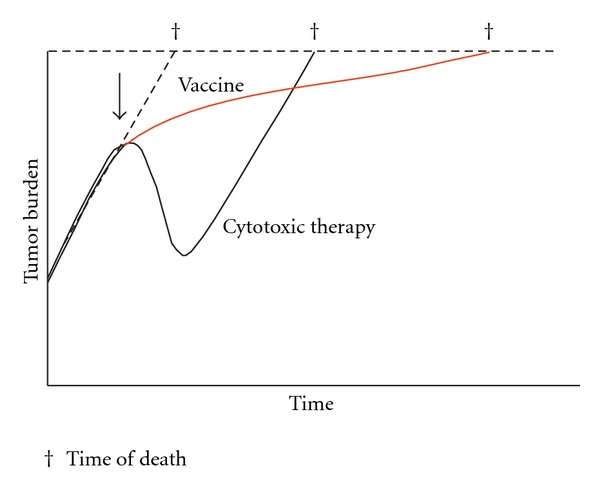
Tumour growth is a dynamic biologic process, that is, the net result of cells dividing and other cells dying. Intrinsic tumour biology, as well as extrinsic factors such as therapies, affects the tumour's growth rate. However, chemotherapy only affects the tumour growth rate while it is being administered, which may result in a dramatic but transient response. Following discontinuation of chemotherapy, the growth rate returns to its pretreatment slope, driven by the underlying biology of the tumour. Immunotherapy (red line), on the other hand, can alter the biology of the host by inducing an active antitumour immune response including a memory response. This may not cause an immediate or dramatic change in tumour burden, but continued cumulative slowing pressure on tumour growth rate, especially if started early in the disease course, which may lead to substantially longer overall survival. The arrow indicates the initiation of treatment; cross indicates time of death from cancer [48]. (Figure used with permission from author.)

Commonly accepted treatment paradigm, however, suggests that treatments should initially decrease tumour volume, which can be measured using CT scan. Also, progression-free survival is increasingly used as an alternative end-point of studies. This seems to be unfortunate for immunotherapy, which may initiate an immune response that ultimately slows the tumour growth rate, resulting in longer survival, but not a decrease in tumour volume on CT or an increased progression-free survival (Figure 3). Future trials are currently planned to investigate these hypotheses; however, clinicians at this moment may need to reconsider how to measure success of their immunotherapeutic approach [48].

## 6. Summary

In conclusion, the role of the immune system in mesothelioma is vast. The tumour uses villainous tricks to evade immune surveillance and harnesses itself against the immune system. Immunotherapy tries to modulate this immune system to strengthen the antitumour effect, which is unfortunately hampered by these defence mechanisms from the tumour. At this moment, MSDCs, TAMs, and Tregs seem to be the key players in this process, but undoubtedly extended research will eventually unravel this complex interplay of cells and will reveal more cell types and/or subtypes. Targeting these defence mechanisms could be the key to fully unleash the potential of immunotherapy. Since several cell types are responsible for tumour survival, probably combination therapy targeting multiple cell types will be necessary. It is thrilling that the immunotherapy has been established in several tumour types as a proven therapy in recent years and that many trials are ongoing with promising results. In mesothelioma, the first steps have been made, and, using the accumulating knowledge, immunotherapy will hopefully prove to be an effective treatment.
